# Assessment of Acute Abdominal Pain in Children Presenting to the Emergency Department: A Retrospective Observational Study

**DOI:** 10.7759/cureus.85914

**Published:** 2025-06-13

**Authors:** Faisal G Almalki, Najeeb Q Alqarni, Maram S Althagafi, Aljory S Al Eid, Ziyad A Badri, Mohammed I Alzahrani, Bsaim A Altirkistani, Asma H AbuGhasham, Malek A Alhnaidi

**Affiliations:** 1 College of Medicine, King Saud Bin Abdulaziz University for Health Sciences, Jeddah, SAU; 2 Department of Pediatric Emergency Medicine, King Abdullah Specialized Children's Hospital, Ministry of National Guard Health Affairs, Western Region, Jeddah, SAU

**Keywords:** abdominal pain, acute abdomen, acute appendicitis, pediatrics, surgical abdomen

## Abstract

Objectives: The aim of the study was to assess acute abdominal pain presentation in pediatric patients presenting to the emergency department.

Methods: This is a retrospective study in which data was collected from electronic medical records. Presentations, associated symptoms, findings on physical examination, documented diagnostic tests, and outcomes of patients were collected and examined. Two multivariate analysis models were used to investigate factors associated with acute medical abdomen and acute surgical abdomen.

Results: Out of 2,169 visits during the study period, 235 (10.8%) patients presented with acute abdominal pain. Forty-five (19%) patients had a surgical abdomen, while the remaining had an acute medical abdomen. The most prevalent diagnosis was "non-specific abdominal pain", constituting 61 (26%) patients. Both leukocytosis (OR: 0.15; 95% CI: 0.03-0.51; p = 0.006) and right lower quadrant pain (OR: 0.26; 95% CI: 0.08-0.81; p = 0.021) were inversely associated with an acute surgical abdomen and were more suggestive of an acute medical abdomen. In contrast, right lower quadrant pain (OR: 13.9; 95% CI: 5.03-43.5; p < 0.001), leukocytosis (OR: 3.26; 95% CI: 1.07-11.1; p = 0.045), and neutrophilia (OR: 1.04; 95% CI: 1.01-1.09; p = 0.031) were found to be significantly associated with acute appendicitis.

Conclusion: Pediatric patients' presentations to the emergency department exhibit a diversity of presentations, and diagnostics may be less evident compared to adult patients. Right lower quadrant pain and leukocytosis may be more indicative of a non-surgical abdomen rather than a surgical abdomen among different diagnoses that require surgical intervention. However, right lower quadrant pain and leukocytosis are still strongly associated with acute appendicitis out of different surgical diagnoses. To combine both findings, our conclusion is that right lower quadrant pain and leukocytosis are more predictive of an acute medical abdomen, but only when appendicitis is less likely to be one of the differential diagnoses. These findings challenge conventional clinical paradigms, suggesting that right lower quadrant pain could be a stronger predictor of medical conditions, which typically do not require surgical intervention.

## Introduction

Acute abdominal pain (AAP) in children is one of the most common reasons for seeking medical care in both general practice (GP) and emergency department (ED). It is also associated with a significant number of hospital admissions and high morbidity rates [1,2]. Since AAP is a common symptom of a wide range of pediatric medical conditions, from self-limiting (e.g., gastroenteritis, constipation, and urinary tract infections) to life-threatening conditions that necessitate immediate surgical intervention (e.g., intestinal obstruction, incarcerated inguinal hernia, testicular torsion, intussusception, volvulus, and appendicitis), it is a challenging complaint to assess and should be entitled to specific attention [3].

The need to recognize an accurate prevalence and to identify different acute abdomen presentations in the ED is established since it will assist clinicians in efficiently diagnosing, managing, and safely discharging patients under better-optimized protocols. Recognizing the true prevalence of acute abdomen cases as well as identifying their varied presentations in the ED is crucial. This enables clinicians to diagnose and manage patients more effectively, implement optimized protocols, and safely discharge patients. As a result, it minimizes the risk of overlooking urgent surgical or medical interventions while also reducing healthcare staff workload, costs, and equipment overuse [3].

As of this review, no local studies have estimated the prevalence or various presentations of AAP in pediatric patients. Thus, this study aims to estimate AAP prevalence in children, evaluate acute abdomen presentations in the pediatric population, and explore factors associated with surgical versus medical causes of abdominal pain in pediatrics.

## Materials and methods

This is a retrospective cohort study conducted in the ED of King Abdullah Specialized Children's Hospital (KASCH), Jeddah, Mecca, Saudi Arabia, after obtaining approval from the Institutional Review Board of King Abdullah International Medical Research Center (approval number: IRB/1799/22). KASCH is a tertiary center with an average of 20,000 visits per year for pediatric patients. The electronic medical record (EMR) software in the hospital is the BESTcare EMR system. We included children aged 3-16 years who were admitted to the ED from May 1, 2021, to June 1, 2022, with a chief complaint of AAP. We excluded children with chronic abdominal pain, immunocompromised patients, and those with a previous history of presentation with abdominal pain during the last two weeks that is related to their current visit. With a confidence interval of 95%, an assumed prevalence of AAP presentation based on our unit audit of 5%, and an assumed precision of 0.01, the estimated sample size was determined to be 1,962 pediatric patients. This study primarily aims to estimate the prevalence of AAP in pediatric patients, with secondary objectives of evaluating acute abdomen presentations and investigating factors associated with surgical versus medical causes of abdominal pain, by assessing key study components including patients' presentations, associated symptoms, physical examination findings, diagnostic tests, and patient outcomes.

An electronic data collection form was distributed to the authors and data were extracted from the EMR system. The data collection sheet was predefined, and all data collectors were medical students. Any conflicts or unclarity about the data extracted was discussed with the pediatric emergency consultant for minimal intra-rater variability. The data collection sheet consisted of six sections: demographic variables (age, sex, weight, and BMI), ED presentation (pain site, duration, quality, character, and radiation), associated symptoms (such as constipation, oliguria, fever, diarrhea, rash, weight loss, bloody stool, testicular pain, hematuria, hematemesis, dysuria, vomiting, bilious emesis, polyuria, dysmenorrhea, jaundice, and shortness of breath), physical examination, diagnostic tests (including abdominal or chest X-ray, ultrasound, abdominal CT, and blood, urine, and stool studies), and diagnosis and disposition. Data was collected in a Microsoft Excel sheet (Microsoft Corp., Redmond, WA, USA), cleaned, and imported to R software (R Foundation for Statistical Computing, Vienna, Austria). Descriptive statistics were used with frequency, and percentages were used for categorical variables, while mean and standard deviation were used for continuous variables. Multivariate regression analysis was used to determine the predictors of acute surgical abdomen and appendicitis. The adjusted R2 was reported. A p-value of <0.05 was considered statistically significant.

## Results

Out of 2,169 visits during the study period, 260 (12%) children were evaluated for AAP. We excluded 25 (1%) patients, resulting in 235 (10.8%) patients' data analyzed. One hundred twenty-five (53%) patients were females, 167 (71%) were 3-9 years old, and 68 (29%) were 10-16 years old. One hundred seventy-four (74%) patients had no significant past medical history, and 151 (64%) patients had at least one abnormal vital sign (see Table 1).

**Table 1:** Demographic characteristics of the study participants *Values are presented as N (%). G6PD: glucose-6-phosphate dehydrogenase

Characteristic	N = 235^*^
Age	
3-9 years	167 (71%)
10-16 years	68 (29%)
Sex	
Female	125 (53%)
Male	110 (47%)
Heart rate	
Bradycardia	7 (3%)
Tachycardia	82 (35%)
Normal for age	146 (62%)
Blood pressure	
Hypotensive	30 (12.7%)
Hypertensive	8 (3.4%)
Normotensive	197 (83.8%)
Respiratory rate	
Normal for age	146 (62.1%)
Tachypnea	89 (37.9%)
Vital signs	
No vital sign abnormalities	84 (35.7%)
At least one abnormal vital sign	151 (64.3%)
Previous history of any medical condition	
No significant past medical history	174 (74%)
Diabetes mellitus	20 (8.5%)
G6PD	5 (2.1%)
Asthma	15 (6.4%)
Chronic constipation	4 (1.7%)
Seizure disorder	4 (1.7%)
Blood disorders	4 (1.7%)
Others	9 (3.8%)

The most frequently reported associated symptoms were vomiting in 137 (58.30%) patients and diarrhea in 68 (28.94%) patients, followed by fever in 54 (22.98%) patients. On physical examination, abdominal tenderness was the most prevalent finding, observed in 127 (54%) patients, with tenderness localized in 74 (58%) of these cases, specifically in the epigastric region in 44 (35%), right lower quadrant (RLQ) in 33 (26%), and umbilical region in 29 (23%) patients. Other clinical findings included rebound tenderness in 24 (10%) patients, guarding in nine (4%) patients, abdominal distension in seven (3%) patients, and a positive McBurney's sign in nine (4%) patients. Pain duration was less than one day in 94 (40%) patients, more than two days in 73 (31%) patients, and one to two days in 68 (29%) patients (see Table 2). At least one abnormal vital sign was recorded in 151 (64.26%) patients, with tachycardia observed in 82 (34.89%) patients and tachypnea in 89 (37.87%) patients being the most common.

**Table 2:** Patient presentation and pain characteristics of the participants *Values are presented as N (%). LUQ: left upper quadrant; LLQ: left lower quadrant; RLQ: right lower quadrant

Characteristic	N = 235^*^
Pain duration	
Less than 1 day	91 (38.7%)
1-2 days	67 (28.5%)
More than 2 days	70 (29.7%)
Not reported	7 (3%)
Timing of pain	
Constant	18 (7.66%)
Not reported	160 (68.09%)
Intermittent	57 (24.26%)
Shifting of pain	
No	54 (22.98%)
Yes	14 (5.96%)
Not reported	167 (71.06%)
Site of pain shifting	
Abdominal to testicular	1 (7.14%)
Epigastric to left flank	1 (7.14%)
Epigastric to LUQ	1 (7.14%)
Generalized then moved to LLQ	1 (7.14%)
Periumbilical to RLQ	7 (50%)
RLQ to LLQ	2 (14.29%)
RLQ to umbilicus	1 (7.14%)
Not reported	221
Quality of pain	
Localized	136 (57.87%)
Diffused	30 (12.77%)
Unspecified	69 (29.36%)
Patient appearance at presentation	
Toxic	19 (8.09%)
Well, active, alert	188 (80%)
Not reported	28 (11.91%)

**Figure 1:**
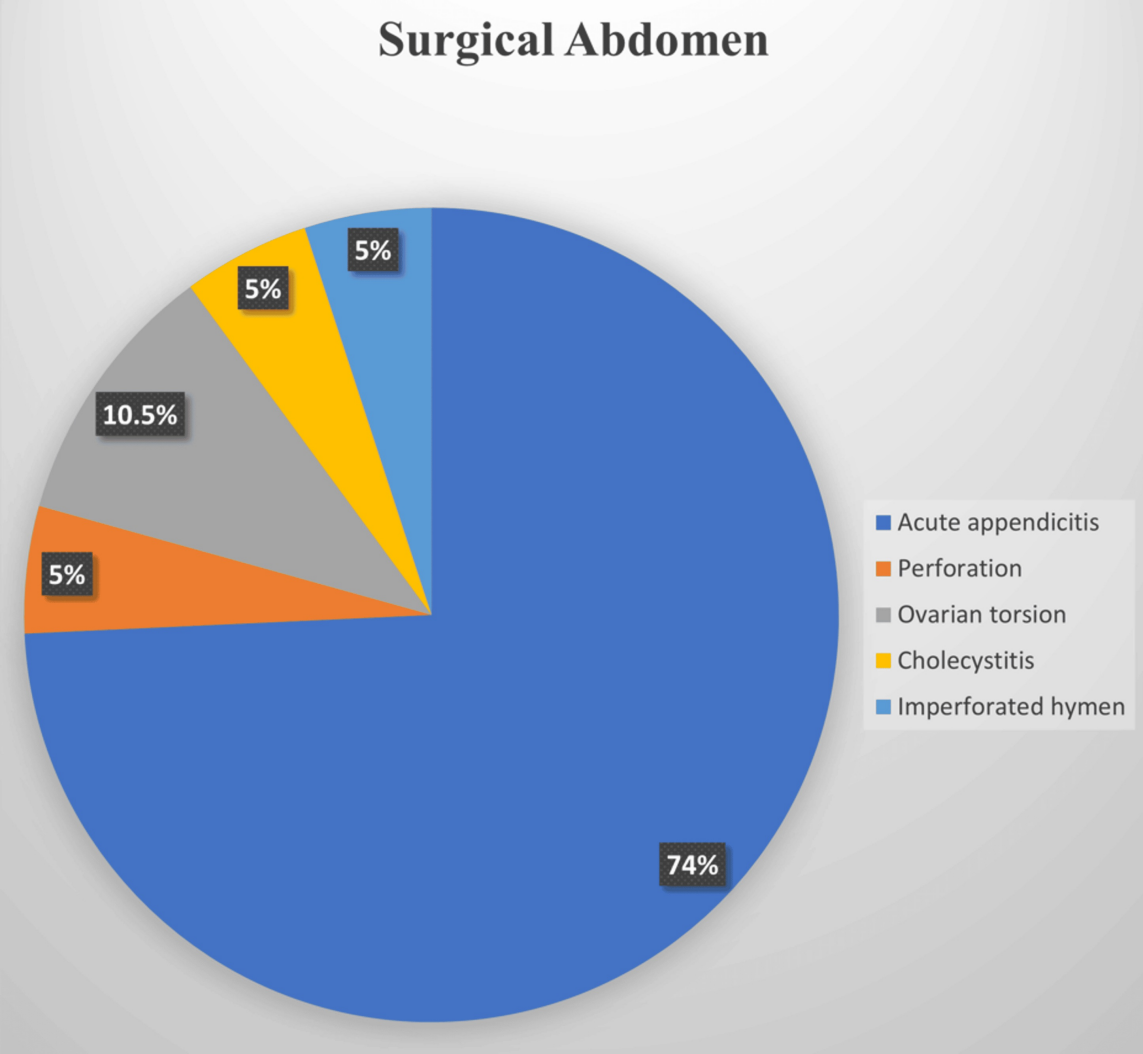
Prevalence of different diagnoses among patients diagnosed to have an acute surgical abdomen

**Figure 2:**
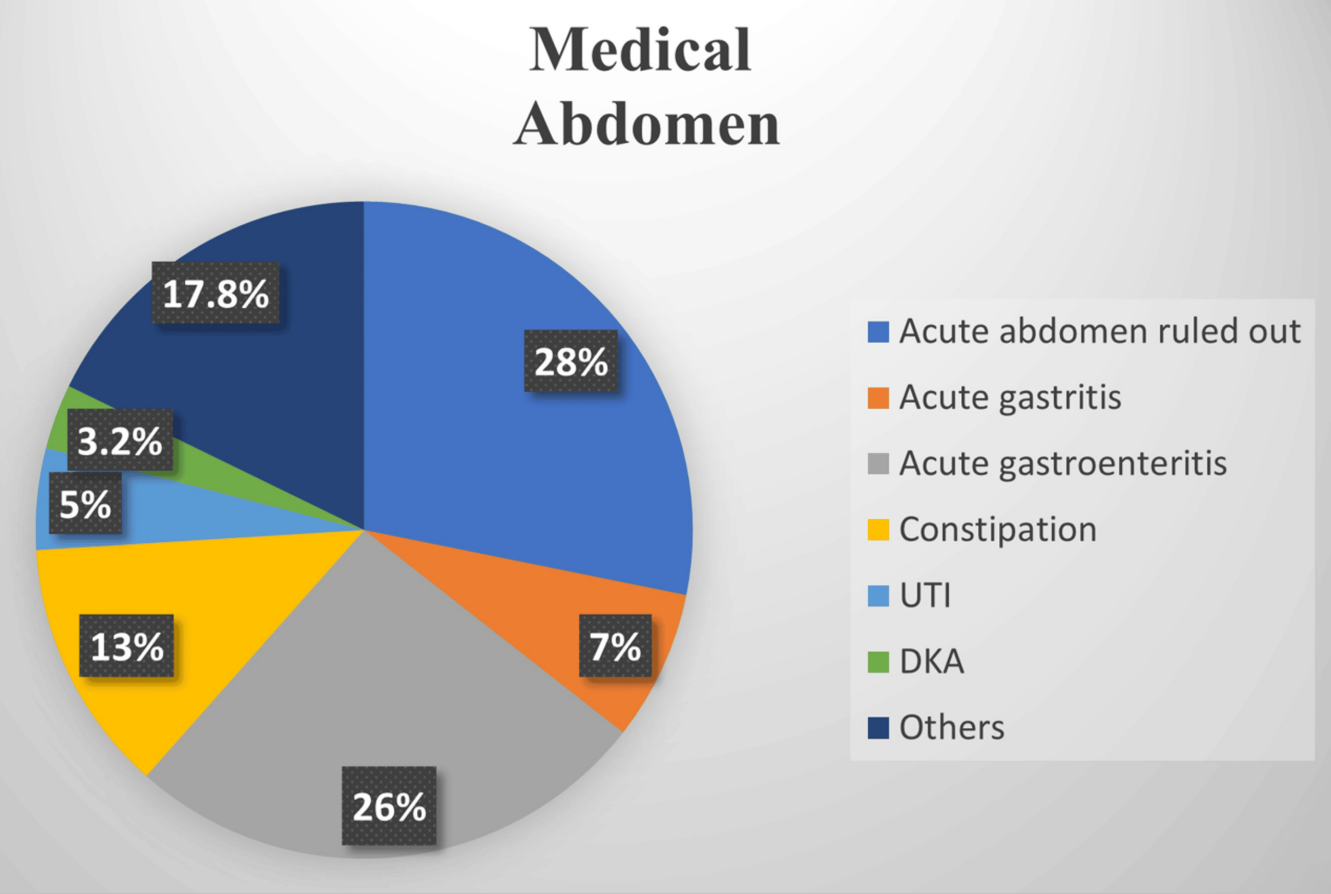
Prevalence of different diagnoses among patients diagnosed to have an acute medical abdomen UTI: urinary tract infection; DKA: diabetic ketoacidosis

Overall, an acute surgical abdomen was diagnosed in 19 (8%) patients, while an acute medical abdomen was diagnosed in 216 (91%) patients. The most prevalent diagnoses of AAP in the surgical abdomen group were acute appendicitis in 14 (74%) patients and ovarian torsion in two (10.5%) patients (see Figure 1). In the medical abdomen group, the most prevalent diagnoses of AAP were "non-specific abdominal pain" in 60 (28%) patients, acute gastroenteritis in 56 (26%) patients, constipation in 28 (13%) patients, acute gastritis in 15 (7%) patients, and urinary tract infection in 11 (5%) patients (see Figure 2).

Leukocytosis was observed in 113 (48%) patients (predominantly neutrophils), and elevated C-reactive protein levels were noted in 120 (51%) patients, representing the most common laboratory findings. Kidney, ureter, and bladder (KUB) X-ray was the most frequently utilized imaging modality, whereas abdominal computed tomography (CT) was the most conclusive modality (see Table 3). One hundred sixty (68%) patients were discharged home from the ED without requiring follow-up. Management in the emergency room with subsequent clinic follow-up occurred in 27 (11.49%) patients, admission to the pediatric medical ward was required in 31 (13%) patients, and 19 (8%) patients were admitted to the pediatric surgical ward.

**Table 3:** Diagnostic images result of the participants *Values are presented as N (%). KUB: kidney, ureter, and bladder; CT: computed tomography

Characteristic	N = 235^*^
Abdominal, pelvic, and inguinal ultrasound	
Normal	20 (8.51%)
Abnormal	43 (18.30%)
Not performed	172 (73.19%)
Abnormal ultrasound result	
Conclusive	16/43 (38.10%)
Inconclusive	26/43 (61.90%)
Abdominal and KUB X-ray	
Normal	48 (20.43%)
Abnormal	21 (8.94%)
Not performed	166 (70.64%)
Abdominal and KUB X-ray results	
Conclusive	16/21 (72.73%)
Inconclusive	6/21 (22.73%)
Chest X-ray	
Abnormal	2 (0.85%)
Normal	22 (9.36%)
Not performed	211 (89.79%)
Chest X-ray results	
Conclusive	2/2 (100%)
Inconclusive	0/2 (0%)
Abdominal CT	
Abnormal	9 (3.85%)
Normal	1 (0.4%)
Not performed	225 (96.1%)
Abdominal CT results	
Conclusive	7 (77.78%)
Inconclusive	2 (22.22%)

Two multivariate analysis models were subsequently used to evaluate factors associated with acute surgical abdomen and acute appendicitis. The first analysis was done to examine the predictors of acute surgical abdomen. Both leukocytosis and RLQ pain were found to be inversely associated with acute surgical abdomen. Specifically, the odds of having an acute surgical abdomen were lower in patients with leukocytosis (OR: 0.15; 95% CI: 0.03-0.51; p = 0.006) and RLQ pain (OR: 0.26; 95% CI: 0.08-0.81; p = 0.021) (Table 4).

**Table 4:** Predictors of acute surgical abdomen and appendicitis among the study participants *A p-value <0.05 indicates statistical significance. Pseudo-R²: 0.23 for acute surgical abdomen predictors and 0.341 for appendicitis predictors. OR: odds ratio; CI: confidence interval; RLQ: right lower quadrant

Outcome	Characteristic	OR	95% CI	P-value
Acute surgical abdomen predictors				
Duration of abdominal pain	Less than 1 day	-	-	-
	1-2 days	0.78	0.21, 3.13	0.7
	More than 2 days	0.75	0.20, 2.89	0.7
RLQ pain	Yes	0.26	0.08, 0.81	0.021*
Vomiting	Yes	0.66	0.16, 2.36	0.5
Fever	Yes	0.87	0.26, 3.20	0.8
Leukocytosis	Yes	0.15	0.03, 0.51	0.006*
Appendicitis predictors				
RLQ pain	Yes	13.9	5.03, 43.5	<0.001*
Pain duration	Less than 1 day	-	-	-
	1-2 days	1.10	0.35, 3.34	0.9
	More than 2 days	0.39	0.09, 1.43	0.2
Leukocytosis	Yes	3.26	1.07, 11.1	0.045*
Neutrophilia	Yes	1.04	1.01, 1.09	0.031*

In the second multivariate analysis, RLQ pain (OR: 13.9; 95% CI: 5.03-43.5; p < 0.001), leukocytosis (OR: 3.26; 95% CI: 1.07-11.1; p = 0.045), and neutrophilia (OR: 1.04; 95% CI: 1.01-1.09; p = 0.031) were found to be significantly associated with acute appendicitis (Table 4).

## Discussion

We describe 235 children arriving at the ED with AAP. The rate we documented, 10.8% of visits, is consistent with a series by Magnúsdóttir et al. from Iceland (12%) but higher than a report by Norbedo et al. from Italy (5.1%) [4,5]. These variations may be attributed to differences in study populations, geographical locations, or diagnostic criteria. One of the notable findings of our study is that 19% of AAP cases required surgical intervention, much higher than in a hospital in New Delhi, India (8%) [6]. This is different from the surgical case rate reported in our study.

In addition to our analysis of AAP cases, a detailed assessment of the demographic characteristics and presentation features of our study participants provides fundamental insights into our patient population. Our cohort predominantly consisted of younger children, with 71% aged between three and nine years. Upon presentation, a significant proportion of our study participants exhibited abnormal vital signs, with 34.89% experiencing tachycardia and 37.87% presenting with tachypnea, underscoring the severity with which acute abdomen can present in pediatric patients. Furthermore, the presentation characteristics revealed diverse symptomatology, as the pain duration among our patients varied significantly. Sheikh and Latif reported in their study in the UAE that 89% of their pediatric patients experienced pain for less than three days [7]. Moreover, our observation that a significant proportion of patients presented without prior medical conditions (74% medically free) suggests that AAP can be a primary concern leading to ED visits among otherwise healthy children.

In our population, the leading causes of AAP were non-specific acute abdomen (26%) and acute gastroenteritis (25%). These results align with other studies reporting non-specific abdominal pain (15.4%) and gastroenteritis (15.4%) as predominant diagnoses [8]. The significant rate of non-specific and undiagnosed acute abdomen accentuates the challenge in diagnosing AAP and suggests an opportunity for refining diagnostic protocols and criteria within pediatric emergency settings [8]. Another explanation for this high rate of non-specific diagnoses could be related to the retrospective nature of the study or poor documentation. Moreover, presenting during the early course of illnesses may hinder the diagnosis as the clinical and biochemical picture may not be completely evident. Acute appendicitis (6%) was consistent with the 4% reported by Mahani et al. in Iran [9]. Furthermore, appendicitis was the most common cause of surgical abdomen in our study (74%), which aligns with the 69% reported by Tseng et al.'s study [10]. This affirms appendicitis as a significant cause of AAP requiring surgical intervention.

Our first multivariate analysis model, aimed at identifying predictors for acute surgical abdomen, revealed an inverse association between acute surgical abdomen and both RLQ pain and leukocytosis. Intriguingly, RLQ pain, which is commonly associated with surgical conditions, was inversely correlated with the likelihood of an acute surgical abdomen [9,11-14]. This suggests that RLQ pain may more frequently indicate a medical rather than a surgical abdomen, particularly when acute appendicitis is excluded as a diagnosis. Although RLQ pain is more commonly correlated with appendicitis, hence surgical abdomen, these findings challenge conventional clinical paradigms, suggesting that RLQ pain could be a stronger predictor of medical conditions such as gastroenteritis, constipation, or urinary tract infections, which typically do not require surgical intervention, especially if it is not associated with elevated white blood cell (WBC) or neutrophil counts [15]. Supporting this, Bundy et al. found in their study that RLQ pain is a stronger predictor of appendicitis in adults compared to children, indicating that RLQ pain may be less indicative of acute appendicitis in the pediatric population [16]. This finding aligns with the Pediatric Appendicitis Score (PAS) and Alvarado Score, which incorporate RLQ pain alongside other markers (e.g., fever, leukocytosis) to enhance specificity for appendicitis [15]. However, as our study is descriptive, this inverse relationship is an observation requiring prospective validation to clarify its clinical significance.

The second model reaffirmed the association of RLQ pain, leukocytosis, and neutrophilia with acute appendicitis, aligning with existing literature that recognizes these factors as traditional markers for appendicitis [12,17,18]. While these observations do not diverge from established knowledge, they validate the utility of these markers in the diagnostic process for appendicitis within our study population.

The utilization of diagnostic imaging plays a significant role in the AAP diagnostic approach. Abdominal and kidney, ureter, and bladder (KUB) X-rays were the most commonly utilized imaging modalities in our study. However, while X-rays were frequently used, the literature generally favors ultrasound for its higher sensitivity and specificity in diagnosing appendicitis [19-21]. Our findings showed that X-rays were particularly effective in our patient population in terms of sensitivity in which they were highly conclusive when clinically indicated. However, the high rate of inconclusive ultrasound results in our study suggests a potential area for improvement in either the technology used or the interpretative skills of clinicians [22-25]. These findings do not support or recommend the utility of abdominal X-ray in pediatric patients presenting to ED with abdominal pain, but rather describe its sensitivity when clinically indicated, especially when perforation is present. 

The findings from our study, particularly the predictive model for acute surgical abdomen, provide new insights that could influence future diagnostic strategies and clinical guidelines. Practitioners should consider a broader spectrum of diagnoses when evaluating pediatric patients with RLQ pain, beyond the traditional suspicion of appendicitis, and even consider extra-abdominal etiologies [10,26,27]. Although we tried to examine any association between different laboratory markers across different etiologies, none of the available markers were helpful, apart from the acute appendicitis markers, which are well-recognized in the literature. In a similar effort to identify relevant markers, Atef Abdelsattar Ibrahim et al. explored in their study the relationship between hemoglobin A1C (HbA1C) and random blood sugar (RBS) in the context of acute surgical abdomen, highlighting their potential as inflammatory markers to guide diagnostic evaluations and inform clinical decision-making [28]. The study concluded that HbA1C levels increase significantly in many cases of surgical acute abdomen, regardless of disease stage, and RBS levels rise with disease progression due to stress hyperglycemia, making them a surrogate marker for inflammation.

The unexpected inverse relationship between RLQ pain and acute surgical abdomen observed in our study suggests a need for further research to understand this association better and its potential implications for clinical practice. However, examining factors associated with a specific etiology may be more promising and valuable. Additionally, exploring the diagnostic accuracy and utility of advanced imaging techniques or biomarkers in AAP could further enhance clinical decision-making and patient outcomes. For instance, integrating more precise imaging methods might reduce diagnostic uncertainty and improve the management of pediatric AAP, ultimately leading to better resource utilization and patient care [10,21,23].

The retrospective nature of our study and the reliance on chart reviews may limit the generalizability of our findings. Furthermore, the absence of prospective validation for our predictive models suggests caution in their application outside a controlled research setting. Future studies should aim to prospectively validate these models in diverse patient populations to ensure their accuracy and applicability in clinical practice.

## Conclusions

This retrospective chart review addresses the longstanding challenge of diagnosing AAP in pediatric patients, offering novel insights into the predictive factors for acute surgical abdomen and reaffirming traditional markers for acute appendicitis. Our findings advocate for a nuanced approach that accommodates both surgical and medical etiologies, potentially shifting clinical assessment paradigms in pediatric AAP. The diverse diagnoses in pediatric AAP, coupled with the non-specific nature of many diagnostic tests, underscore the need for clinicians to enhance clinical evaluation using scientifically grounded methods, such as validated clinical decision tools, to improve diagnostic accuracy. Thorough history-taking and physical examination remain the cornerstone of this process, guiding the differentiation of surgical and medical etiologies with precision.
